# Numerical model of human head phantom to ensure dosimetry of dose components for boron neutron capture therapy

**DOI:** 10.1093/rpd/ncad158

**Published:** 2023-10-11

**Authors:** Edyta Michaś, Katarzyna Tyminska, Michal A Gryzinski, Janusz Kocik, Ryszard J Barczynski

**Affiliations:** Radiological Metrology and Biomedical Physics Division, National Centre for Nuclear Research, Otwock 05-400, Poland; Faculty of Applied Physics and Mathematics, Gdansk University of Technology, Gdańsk 80-233, Poland; Radiological Metrology and Biomedical Physics Division, National Centre for Nuclear Research, Otwock 05-400, Poland; Radiological Metrology and Biomedical Physics Division, National Centre for Nuclear Research, Otwock 05-400, Poland; Radiological Metrology and Biomedical Physics Division, National Centre for Nuclear Research, Otwock 05-400, Poland; School of Public Health, Center of Postgraduate Medical Education, Warsaw 01-826, Poland; Faculty of Applied Physics and Mathematics, Gdansk University of Technology, Gdańsk 80-233, Poland

## Abstract

Extremely important aspects of the boron neutron capture therapy are, first of all, administering to the patient a boron compound that selectively reaches the neoplastic cells, and in the second step, the verification of the irradiation process. This paper focuses on the latter aspect, which is the detailed dosimetry of the processes occurring after the reaction of thermal neutrons with the boron-10 isotope. The results of computer simulations with the use of a new type of human head phantom filled with a polymer dosimetric gel will be presented in this article.

## Introduction

Boron neutron capture therapy (BNCT) is a therapy that provides an opportunity for patients with head and neck, brain and metastatic cancers, which are ineligible for treatment with conventional treatment techniques, and provides a solution when conventional techniques fail to produce the expected results. Concerning current treatment methods, BNCT brings fewer adverse events to patients undergoing treatment, therefore is better tolerated during it and assures a better quality of life afterward. The first stage of therapy involves administering a boron compound to the patient along with a carrier that selectively reaches the tumor cell. In the second stage, the patient is irradiated with a beam of epithermal neutrons. The therapy is based on the nuclear capture and fission reaction of the stable isotope of ^10^B, which after irradiation with thermal neutrons (thermalization of neutrons from epithermal to thermal occurs on the path from the skin to organs located deeper in the body) produces a high-energy alpha particle and a ^7^Li recoil nucleus, which may be excited and emit a gamma quantum. The described reaction occurs at the cellular level. Correct dose determination plays a very important role in the therapeutic process. The total dose in BNCT consists of four components: high-linear energy transfer (LET) boron dose, neutron dose, the proton dose from protons emitted in nitrogen capture reaction and low-LET gamma dose^(^^1^^)^. One important element in optimizing the therapeutic effect of BNCT is the correct determination of the components of the dose.

## Project objective

The goal of this project is to prepare a human head phantom along with a dosimetry protocol that will enable the determination of the dose components shown below:

(1) protons coming from the ^14^N (n, p) ^14^C reaction;
(2) protons from fast neutrons scattering;
(3) α-particles and Li recoil nuclei from the ^10^B (n, α) ^7^Li reaction;
(4) gamma rays from the ^1^H (n, γ) ^2^D reaction and from the reactor core^(^^1^^)^.

Work on the project is divided into two main phases. The first of them is strongly related to the phantom construction and restrictions for the material used, whereas the second one is related to the development of a technique for irradiating the prepared phantom at the stand for boron neutron therapy. The Monte Carlo simulations support all the stages of the investigation, from material selection to irradiation in neutron, gamma and mixed radiation fields.

The material that will simulate bone tissue must exhibit:

(a) conformity of all active cross sections for interactions with neutrons and photons to the active cross sections in real bone tissue;
(b) transparency and a relatively low refractive index in the spectral range used in optical tomography;
(c) zero permeability to molecular oxygen whose concentration in the gel must be stable over long time intervals.

**Figure 1:**
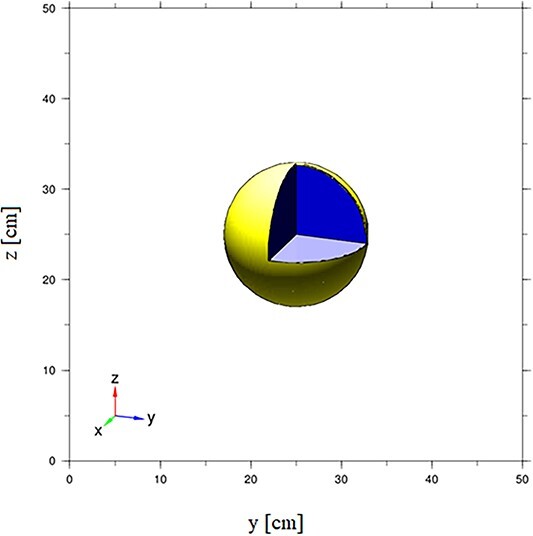
Model of the human head phantom, designed for BNCT.

**Table 1:** The composition of the acrylic glass used in the simulation. The density is 1.136 g cm^−3^.

Elements	W/w fraction
C	0.599848
H	0.080538
O	0.319614

The most important principles associated with the phantom are summarized below:

(a) the human head has been approximated as a sphere 23 cm in diameter;
(b) it should be equipped with a system allowing immobilization in a position corresponding to that of the patient’s head during a BNCT irradiation session, in the appropriate position, precision and recurrence;
(c) it will be made of a material that does not activate in a neutron beam.

The first stage of the project involved performing Monte Carlo simulations for phantoms made of different materials and selecting the most optimal solution.

## Methods

The research object is a human head phantom filled with a polymer gel dosemeter. In the first simulations, the approximation of a sphere with a diameter of 23 cm was used. The surface of the sphere was 0.2 cm thick. The model of the sphere phantom is shown in Figure 1. The surface of the sphere was made of three different materials: acrylic glass, quartz glass and borosilicate glass. The chemical composition of used acrylic glass, borosilicate glass and quartz glass is presented in Tables 1, 2 and 3. The sphere made of each of two types of glass was filled with either water or polymer gel dosemeter. The elemental composition of the gel is presented in Table 4.

**Table 2:** The composition of the borosilicate glass used in the simulation. The density is 2.23 g cm^−3^.

Elements	W/w fraction
B	0.040064
O	0.539562
Na	0.028191
Al	0.011644
Si	0.377220
K	0.003321

**Table 3:** The composition of the quartz glass used in the simulation. The density is 2.648 gcm^−3^.

Elements	W/w fraction
Si	0.467435
O	0.532565

**Table 4:** The composition of the gel used in the simulation. The density is 1.08 g cm^−3^^(^^2^^)^.

Elements	W/w fraction
C	0.144085
H	0.100852
N	0.024370
O	0.730688
Cu	3.20 × 10^−7^
S	1.76 × 10^−6^
Fe	2.79 × 10^−6^

**Figure 2:**
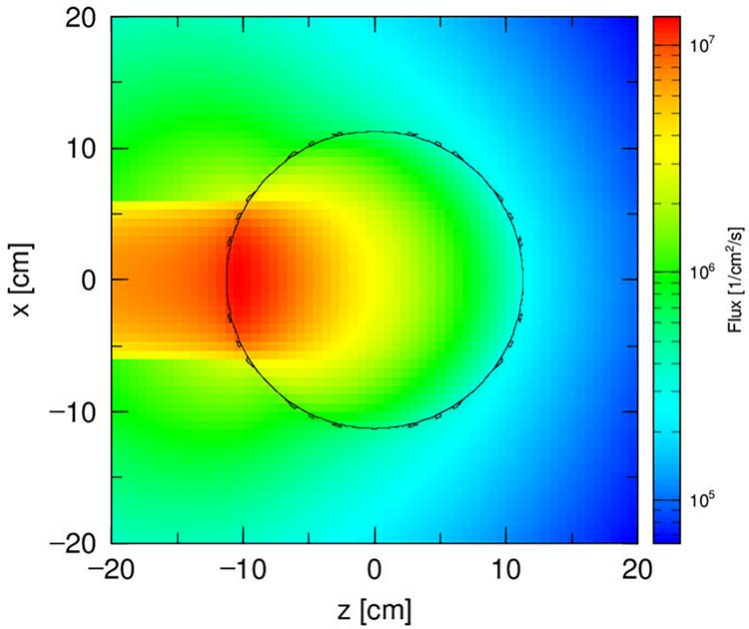
Spatial distribution of the beam from the H2 horizontal channel.

The modeled source energy spectrum corresponds to the beam at the output of the H2 horizontal channel at the MARIA research reactor calculated using a detailed model of the reactor^(^^3^^)^. This channel outputs the neutron beam directly from the core of the MARIA reactor. The spatial distribution of the beam from the H2 horizontal channel is shown in Figure 2.

**Figure 3:**
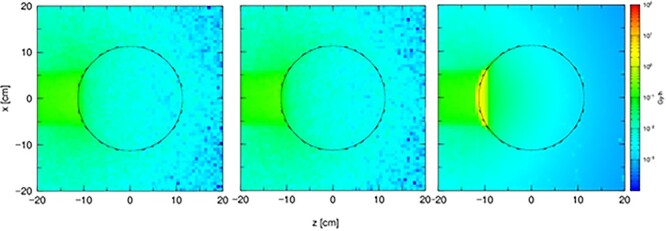
The total dose distribution in a 23 cm diameter sphere filled with water. The sphere is made of (**a**) acrylic glass, (**b**) quartz glass and (**c**) laboratory glass.

**Figure 4:**
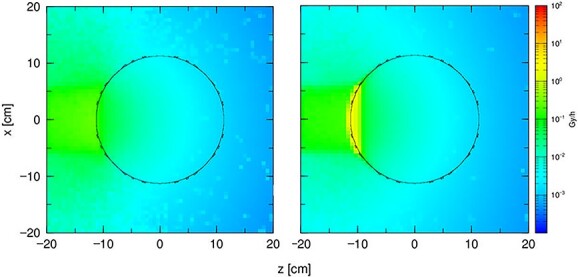
The total dose distribution in a 23 cm diameter sphere filled with the gel dosemeter. The sphere is made of (**a**) quartz glass and (**b**) laboratory glass.

All Monte Carlo simulations were made in Particle and Heavy-Ion Transport code System^(^^4^^)^. The simulation will be extended with a phantom that is compatible with the shape of the human skull. In all of the simulations, default cross sections were used.

## Results

In the first step, simulations employed a sphere filled with water. The acrylic glass water phantom represents the simplest model, which will serve as a reference against the quartz glass phantom filled with polymer dosimetry gel in further simulations and dosimetry measurements. The sphere is placed in front of the beam, in an environment of atmospheric air. The results of the total dose distribution are shown in Figure 3.

In the second step, the sphere is filled with the polymer gel dosemeter. Acrylic glass does not fulfill the requirements to be used as a skeleton for a gel-filled phantom. In addition, it is characterized by too high porosity for a polymer dosimetry gel. The sphere is placed against the beam, in an environment of atmospheric air. The results of the total dose distribution are shown in Figure 4.

**Figure 5:**
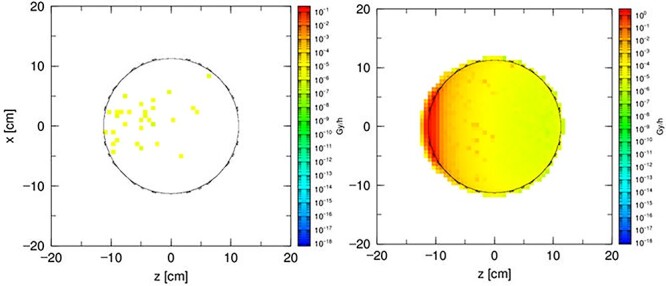
The dose distribution from the reaction boron with the thermal neutrons in a 23 cm diameter sphere filled with a gel dosemeter. The sphere is made of (**a**) quartz glass and (**b**) laboratory glass.

It is necessary to pay special attention to the cross section of the material for the reaction with the neutron beam. Laboratory glass is commonly used in polymer gel dosimetry in different types of radiation fields, but in the neutron field, the boron included in the material reacts with neutrons. The Monte Carlo simulation results for a phantom filled with polymer dosimetry gel are shown in Figure 5.

The above-mentioned results suggest the use of quartz glass as a material for the skeleton of a phantom for BNCT.

## Conclusions

The simulations show different dose distributions for different sphere surface materials using the same radiation source. They will be repeated using a boron solution and a mixture of gel and boron. In the next step, experimental verification of the assumptions and simulation results will take place.

A final result of the work will be a phantom filled with dosimetry gel, allowing the accurate dosimetry of therapeutic dose components at the research stands for BNCT in National Centre for Nuclear Research. The separation of dose components arising because of different LET values of the generated radiation will be possible.

The presented research is a part of the material for a PhD thesis within the Ministry of Education and Science PhD program, the 4th edition.

## Data Availability

Data are available upon reasonable request.
